# A New De Novo Missense Variant of the *TET3* Gene in a Patient with Epilepsy and Macrocephaly

**DOI:** 10.3390/ijms25179676

**Published:** 2024-09-06

**Authors:** Miryam Rosa Stella Foti, Maria Giovanna Tedesco, Davide Colavito, Daniela Rogaia, Amedea Mencarelli, Monica Schippa, Cristina Gradassi, Rita Romani, Carmela Ardisia, Paolo Prontera

**Affiliations:** 1 Unità di Genetica Medica, Azienda Ospedaliera Sant’Anna, Dip. Scienze Mediche e Dip. Materno-Infantile, 44121 Ferrara, Italy; 2 Istituto Malattie Rare “Mauro Baschirotto”, Costozza di Longare, 36100 Vicenza, Italy; 3 UOSD Genetica Medica e Malattie Rare, Dip. Materno-Infantile, Azienda Ospedaliera di Perugia, 06129 Perugia, Italyrita.romani@unipg.it (R.R.);; 4 Research & Innovation (R&I Genetics) Srl, 35100 Padova, Italy; 5 Department of Medicine and Surgery, University of Perugia, 06132 Perugia, Italy

**Keywords:** *TET3*, epilepsy, macrocephaly, dysarthria

## Abstract

The etiology of neurodevelopmental disorders and epilepsy is very heterogeneous and partly still unknown, and the research of causative genes related to these diseases is still in progress. In 2020, pathogenic variants of the *TET3* gene were associated with Beck–Fahrner syndrome, which is characterized by neurodevelopmental delay, intellectual and learning disabilities of variable degree, growth abnormalities, hypotonia and seizures. Variants of *TET3* have been described having both an autosomal dominant with a milder phenotype and an autosomal recessive pattern. To date, in the literature, only 28 patients are reported with pathogenic variants of the *TET3* gene, and only 9 of them have epilepsy. We describe a 31-year-old woman with macrocephaly, mild neurodevelopmental delay and a long history of epilepsy. Trio-based exome sequencing identified a de novo heterozygous *TET3* variant, c.2867G>A p.(Arg956Gln), never described before, absent in the general population and predicted to be potentially pathogenetic by bioinformatics tools. This report aims to describe the clinical history of our patient, the pharmacological treatment and clinical response, as well as the biological characteristics of this new variant.

## 1. Introduction

*TET3* (tet methylcytosine dioxygenase 3), located on chromosome 2p13.1, is a gene involved in epigenetic chromatin reprogramming in the zygote, embryogenesis and neuronal differentiation. Beck et al. [1] described the first cohort of patients (eight families, 11 individuals) with recurrent clinical characteristics and pathogenic variations in the *TET3* gene. Of interest, five patients had biallelic pathogenic variants inherited from healthy parents, and three of them were siblings from a consanguineous family. Other patients had a pathogenic variant in the heterozygous state, and only in one case the variant was inherited from the affected father. The variants described were missense, frameshift and truncating, suggesting loss of function as the mechanism of disease and complete penetrance; no genotype–phenotype correlations were observed. To date, a total of 28 patients have been collected and delineated as affected by *TET3*-related Beck–Fahrner syndrome (*TET3*-BEFAHRS, OMIM #618798) [1,2,3]. Here, we describe a woman of 31 years of age with a history of epilepsy, moderate developmental delay and macrocephaly, who asked for a new genetic counseling to understand her reproductive risk.

## 2. Case Presentation

Our patient is the third child of non-consanguineous and healthy parents and was born at 39 weeks of gestation after an uneventful pregnancy with caesarean section. Her neonatal parameters were 36.5 cm for the head circumference (75–90th centile) and 3.4 kg of weight (50th centile). Perinatal adaptation was without complications, as well as neonatal screening. The patient had a motor delay: her mother reported her first steps at 20 months but an unstable gait until 3 years; about language development, she had a slow acquisition of words.

**Figure 1:**
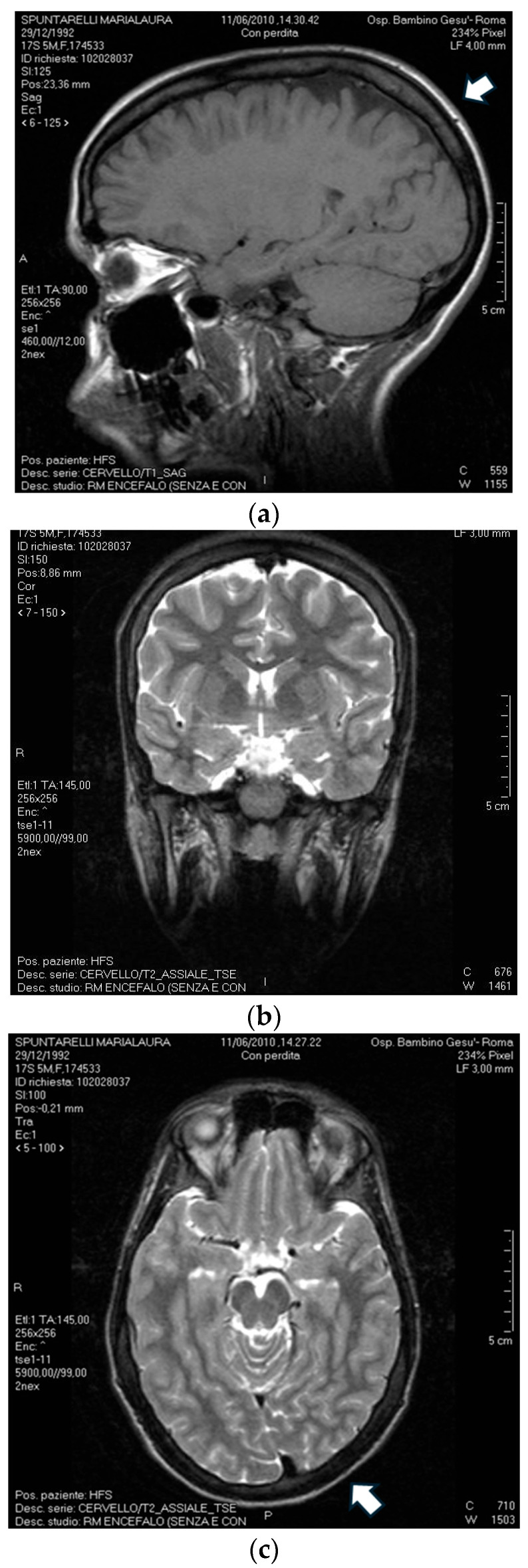
Brain MRI of the proband performed at 18 years of age, with (**a**) no significant malformation in the sagittal plane, white arrow indicates the small expansion of the periencephalic liquor spaces (**b**) asymmetry of the occipital lobes in the coronal plane (**c**) white arrow indicates the asymmetry of the occipital lobes in the axial plane.

At 32 months old, she had a first generalized tonic–clonic seizure that required hospitalization for much of the crisis and was treated with rectal diazepam. Cerebral MRI was performed for the first time at 3 years and showed no significant malformation; only an asymmetry of the occipital lobes (with right prevalence) and a small expansion of the periencephalic liquor spaces were reported and confirmed on the following MRI at 1.5T (Figure 1). In consideration of the motor delay and seizures, variious biochemical, molecular and instrumental exams were required. The values of plasma medium, very long chain fatty acids, urinary organic acids and apolipoproteins were in the normal range. From a neurological point of view, the EEG showed a sharp- and slow-wave complex in the left parieto-temporal region. At this time, therapy was based on phenobarbital per os, with an apparent control of the epileptic manifestation, although with the same EEG pattern. Following an initial state of wellness, ataxia and dysarthria became evident and were described on neurological evaluation. A physical examination at 39 months reported macrocephaly (head circumference of 55 cm, +2SD) and length of 95 cm (50th centile).

**Figure 2:**
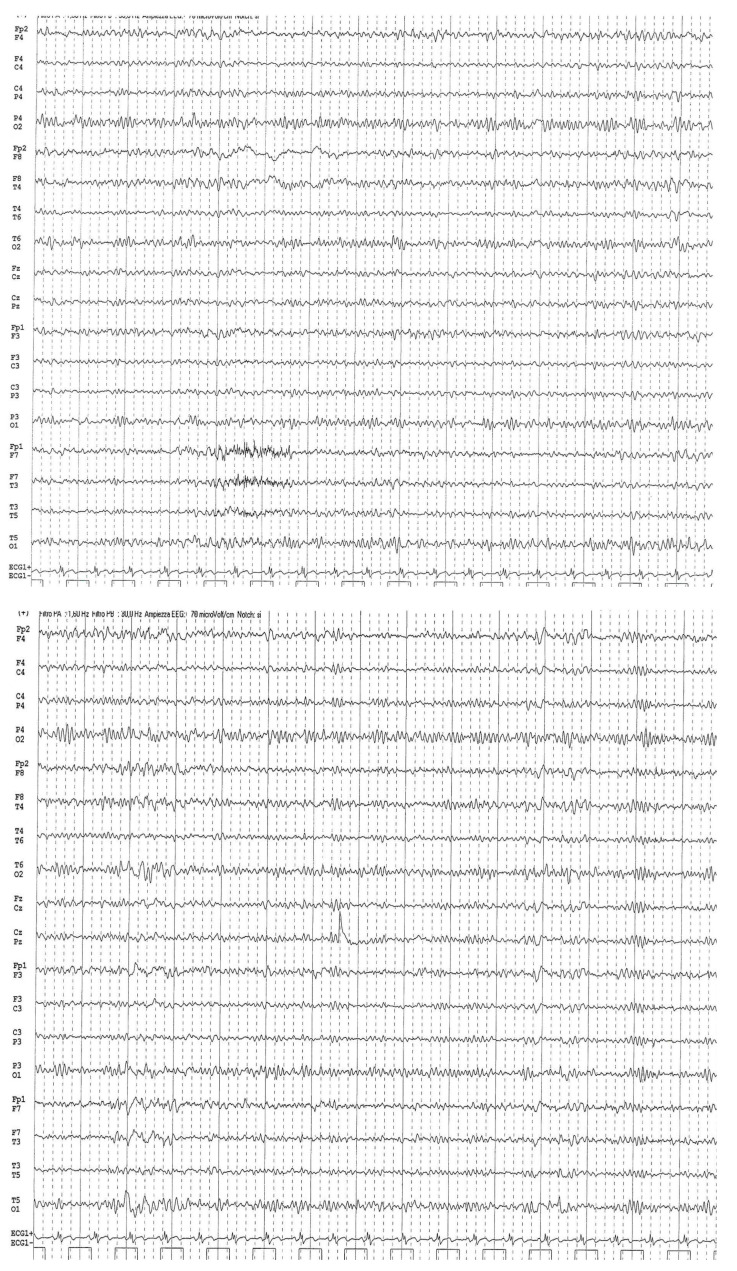
A routine surface EEG of the patient at 14 years old, using the 10/20 international system, in bipolar montage shows electrical status epilepticus during slow-wave sleep (ESES).

Focal seizures were present only during sleep and characterized by a lateral deviation of the mouth. Clinicians carried out numerous EEGs that showed electrical status epilepticus during slow-wave sleep (ESES) (Figure 2). Finding the correct therapy to obtain control of the seizures was challenging. The neurologists started with phenobarbital at the maximum dosage but without achieving control, despite the introduction of a polypharmacologic treatment (carbamazepine, clobazam, vigabatrin, valproate and lamotrigine). It is important to underline that lamotrigine was rapidly uninterrupted because of collateral effects such as marked sedation, asthenia and loss of coordination. Only after eight years of age, a satisfactory drug combination was defined, but our proband achieved full seizure control only with the use of ethosuximide, which allowed for dose escalation and interruption of the other drugs.

At 10 years old, a neuropsychiatric evaluation was performed, which identified a mild cognitive delay, with poor abstraction capacity, attention deficit hyperactivity disorder (ADHD), repetitiveness of learned patterns. However, with support at school and speech therapy, the patient achieved graduation. She showed only a mild delay in physical and puberal development: a metacarpal radiography, performed at 7 years and 11 months of age, was compatible with 6 years and 10 months of skeletal age, and a mild delayed menarche was reported at 15 years of age.

From 14 years of age, she has not had seizures during sleep, but a normal EEG was only detected at 16 years of age, and total suspension of drugs was achieved at 24 years of age.

The first genetic approach included karyotyping, performed on lymphocytes from peripheral blood, which showed a normal karyotype formula (46, XX), as well as the analysis of FRAXA and FRAXE fragile sites. A skin biopsy from the deltoid region excluded granular osmiophilic deposits and, consequently, quite frequent storage diseases. A methylation study of locus 15q11-12 ruled out abnormalities. She continued to undergo neurological follow-ups, without showing other clinical problems.

Only at 22 years old, genetic counselling was requested. The auxological parameters in this first genetic evaluation showed a tall stature, macrocephaly and normal weight, in the context of which some genetic investigations were carried out. At first, array-CGH was performed as previously described [4] and showed a microdeletion of 272 kb on chromosome 11p12, maternally inherited, that we ruled out as causative. As for macrocephaly and seizures, analysis of the *NSD1* gene (related to Sotos syndrome) and a 109-gene panel associated with epilepsy was conducted, without evidence of pathogenic or unknown-significance variants (VoUS). In 2019, a trio-based multigene panel for macrocephaly and developmental delay showed in the patient two variants of unknown significance in compound heterozygous state, each inherited from a parent, in the *LRP2* gene, whose mutations are causative of Donnai–Barrow syndrome (DBS, OMIM **#** 222448). Both variants are absent in population databases and were not previously described in other patients. So, in order to acquire more information and try to clarify the clinical significance of the *LRP2* variants, we proposed a segregation analysis in the patient’s healthy siblings, who resulted heterozygous for only one of the *LRP2* variants. Also, we suggested the following clinical evaluations to look for the presence of other clinical signs attributable to the syndrome: an ophthalmology assessment, audiometry and electrophoresis of urinary proteins (to identify retinol- and vitamin D-binding protein). The ophthalmology assessment with fundus oculi examination was normal. Audiometry revealed a mild conductive bilateral deafness but without impairment of daily activities. Unfortunately, the electrophoresis of urinary protein, which is specific for the condition, could not be carried out. Even if the association of macrocephaly, development delay, deafness and familial segregation of *LRP2* variants could suggest the contribution of the two variants to the phenotype in the patient, she did not show the typical facial dysmorphisms of DBS (such as enlarged globes and prominent eyes, downslanting palpebral fissures, underorbital skin creases, short nose, flat nasal bridge), ocular anomalies (high myopia, iris coloboma/hypoplasia, cataract) or severe intellectual disability. Anyway, we suggested a new genetic counselling a few years later.

In 2023, the patient asked for new counselling in prevision of a possible pregnancy. Regarding her clinical history, although she had not assumed drugs since about ten years of age, she continued to undergo an annual neurological follow-up, without evidence of abnormalities on EEG. During the last neurological evaluation, the EEG showed some slow waves in the frontal lobes during hyperpnea, without clinical manifestations. At this moment, no pharmacological treatment has been recommended.

Considering the absence of an etiologic diagnosis, we asked the same laboratory that performed the multigene panel assay for macrocephaly to extend the analysis to the clinical exome.

**Figure 3:**
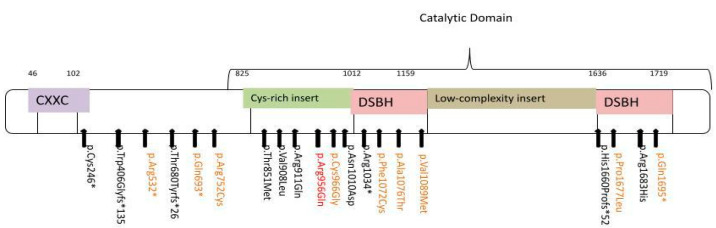
Schematic presentation of TET3 protein structure and pathogenic variants previously described; in orange, variants in patients with an epilepsy phenotype, in red the novel variant. Adapted from Sager et al. [2].

Trio-based exome sequencing identified in the patient the variant c.2867G>A p.(Arg956Gln) in the *TET3* gene in the heterozygous state, which was absent in both parents and siblings. This variant was reported by the laboratory as a variant of unknown significance (VoUS) because it is absent in population databases and was never described in other patients; however, it is located in a conserved locus in different species, and in silico tools such as Mutation Taster, DANN, PrimateAI, BayesDel and GenoCanyon provided contrasting results, although mostly indicative of a possible damaging effect. In the proximity of our variant, other pathogenic missense variants have already been reported, of which 11 are missense, 5 are nonsense, and only 3 are frameshift (Figure 3). In consideration of the clinical characteristics and molecular data, we suggest that the heterozygous missense variant in the *TET3* gene could be causative of the patient’s phenotype.

## 3. Discussion

*TET3*-related Beck–Fahrner syndrome (*TET3*-BEFAHRS) is a recently defined disease characterized by clinical variability, complete penetrance and loss-of-function variants in the *TET3* gene, as heterozygous as well as biallelic variations. Its clinical manifestations are predominantly mild to severe impaired intellectual development, development delay, behavioral psychiatric manifestations (such as autism, ADHD, depression, anxiety), neurological problems, macrocephaly in some patients and movement disorders. Development delay is the predominant feature, reported in almost all patients, whereas clinical evaluations for the other clinical problems were not performed in the 28 reported patients; so, we cannot exclude abnormalities. According to the available clinical data, 56.5% (13/23) of the patients had hypotonia, about 37.5% (9/24) had seizures (variable type), while other recurrent manifestations were a predominantly conductive hearing loss and ophthalmic problems (strabismus, nystagmus and refractive errors); only a minor part of the patients had congenital anomalies (particularly, heart defects). Patients were variably described with growth abnormalities (47%, 9/19), most of them showing overgrowth (6/19, 4 of which had macrocephaly), but some showing undergrowth and microcephaly (3/19). A fraction of the patients, about 47% (9/19), reported musculoskeletal findings such as joint hypermobility (4/9), hip dysplasia (3/9), kyphosis and/or scoliosis (2/9), and one patient had an inguinal hernia (Table 1). The facial features are not specific and were described as long and myotonic face with a tall and/or broad forehead and protruding ears; the ocular region was characterized in some patients by epicanthal folds, thick or arched eyebrows and downslanted palpebral fissures, while the nose could be short with a long philtrum.

**Figure 4:**
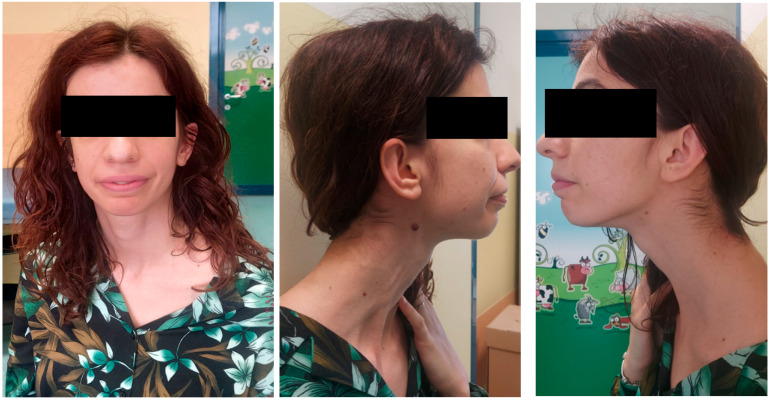
Facial appearance of the patient at the age of 32 years: tall and broad forehead, long face, epicanthal folds, arched eyebrows, simplified ears, broad nose with smooth philtrum.

In our patient, the overlapping features with those of *TET3-BEFAHRS* were development delay, in particular, speech, gross motor and fine motor delay, mild intellectual disability, tall stature (height above the 97th centile), macrocephaly (OFC 61 cm), normal weight (3–25th centile), movement disorder (ataxia), scoliosis, the pattern of ESES, which was described in two other patients as a unique pattern associated with a crisis, and seizures refractory to standard therapy, as in our patient [1,2] and in one patient with complex partial epilepsy [3]. The facial features are compatible, even if not specific, with *TET3-BEFAHRS*. In fact, our patient has a tall and broad forehead, a long face, epicanthal folds, arched eyebrows. However, she has some morphological peculiarities: simplified ears, a long neck, a broad and long nose with a smooth philtrum and, from a neurological point of view, speech difficulties, characterized by oral–buccal–facial dyspraxia (Figure 4).

## 4. Conclusions

Our report demonstrates that it is mandatory to re-evaluate undiagnosed or dubious cases to promote continuous progress in scientific knowledge and achieve correct diagnoses. Moreover, detailed descriptions of patients with recently described syndromes help to identify clinical elements for disease diagnosis and could expand the number of the related phenotypes. We had the possibility to describe the drugs used and their clinical benefit, which could help clinicians to choose a successful therapy for patients with a new diagnosis of *TET3*-BEFAHRS.

Additionally, we described a novel gene variant that is still classified as VoUS only for lack of knowledge but we added strong clinical evidence to support its pathogenicity.

The description of an adult patient who asked for a precise diagnosis in pre-conception counseling is really important for other families that receive a diagnosis of *TET3*-BEFAHRS, as it allows for understanding the clinical variability of the disease and also the possible good quality of life and prognosis of patients.

## Figures and Tables

**Table 1:** This table shows the main characteristics of the patients described in the literature compared with those of our patient.

Feature	Proportion of Persons with Features of TET3-BEFAHRS	Our Patient
Developmental delay and/or intellectual disability	25/26	Yes
Social communication disorder	11/13	No
Autistic features/autism spectrum disorder	9/14	No
Anxiety	8/11	NA
Hypotonia	13/23	No
Hearing loss (predominantly conductive)	7/10	Yes (conductive)
Musculoskeletal findings (joint hypermobility, hip dysplasia, scoliosis/kyphosis)	9/19	Yes (scoliosis)
Attention-deficit/hyperactivity disorder	6/13	Yes (ADHD)
Growth abnormalities	9/19 6/19 overgrowth 3/19 undergrowth.	Yes (overgrowth)
Gastrointestinal manifestations (feeding difficulties and/or constipation)	8/18	NA
Ophthalmologic findings (refractive errors, strabismus, nystagmus)	9/22	No
Seizure disorder	9/24	Yes (ESES)
Other abnormal movements (tics, myoclonic jerks, dysmetria, posturing and dystonia)	7/23	No
Congenital heart defects (valve abnormalities or complex congenital heart disease)	5/19	No

NA: not applicable, ESES: Electrical Status Epilepticus During Slow-wave Sleep. Adapted from Adam et al. [5].

## Data Availability

The data presented in this study are available on request from the corresponding author.
